# The effect of a terrorist attack on emergency department inflow: an observation study using difference-in-differences methodology

**DOI:** 10.1186/s13049-019-0634-2

**Published:** 2019-05-24

**Authors:** Andreas Ekström, Fredrik Eng-Larsson, Olov Isaksson, Lisa Kurland, Martin Nordberg

**Affiliations:** 1 Department of Clinical Science and Education, Södersjukhuset, Karolinska Institutet, Stockholm, Sweden; 2 Stockholm Business School, Stockholm University, Stockholm, Sweden; 3 Department of Medical Sciences, Örebro University, Örebro, Sweden; 4 Department of Emergency Medicine, Örebro University Hospital, Örebro, Sweden

**Keywords:** Emergency service, Hospital, Patient acceptance of health care, Terrorism, Health behavior

## Abstract

**Study objective:**

The objective of this study was to investigate how the terrorist attack in Stockholm, Sweden affected patient inflow to the general emergency departments (EDs) in close proximity of the attack. The study analyzed if, and to what extent, the attack impacted ED inflow during the following days and weeks.

**Methods:**

In a retrospective observational study, anonymized aggregated data on ED arrivals (inflow of patients) to all seven of the EDs in the Stockholm County was analyzed using the Difference-in-Differences (DiD) estimator. The control groups were the affected hospitals in the years prior to the terrorist attack. The number of ED visits was retrieved from the Stockholm County Council administrative database.

**Results:**

The study shows a statistically significant reduction in overall ED inflow of 7–9% following the attack. The effect was strongest initially after the attack, and ED inflow regained normal levels within approximately three weeks’ time, without any significant rebound effect. The effect on ED inflow also decreased with distance from ground zero, and was not significant further away than 10 km.

**Conclusion:**

The results showed that ED inflow was significantly decreased in the weeks immediately following the Stockholm terrorist attack. The reasons for this cannot be fully explained in this observational study. However, the results suggest that some patients actively choose when, where and if they should go to the ED.

## Background

At 2:43 pm, Friday, April 7th 2017 a single terrorist drove a delivery truck down one of the busiest shopping streets of Stockholm, Sweden. Five people were killed and 14 were severely injured during the truck’s short campaign, which abruptly ended with the truck crashing into the front of a major department store [1, 2].

In the past, terrorist attacks have been shown to have a large immediate impact on the inflow of patients to emergency departments (EDs). E.g. more than 1000 victims were treated in the five closest hospitals during the first 48 days after the terrorist attack on the World Trade Center in 2001 [3]. While many victims were seriously injured, over-triage was also reported. I.e. minimally injured victims received a high triage priority and were sent to the hospitals despite the lack of medical necessity [4], a problem observed during other terrorist attacks as well [5–7]. During and immediately after a terrorist attack with many victims EDs in the close proximity will be burdened, both by severely and less injured.

Terrorist attacks also affect the ED inflow on the longer term for specific symptoms and diagnoses, even for people not directly injured in the attack. While some people act bravely and philanthropically in the community right after an attack, acute stress reactions are common [4]. Again, exemplified by the attack on the World Trade Center, there was a 10% increase in behavioral and mental health diagnoses in the EDs in close geographical proximity to the attack [8]. In addition, media plays a role in affecting the longer-term inflow. Previous studies have found that media coverage of adverse events influences healthcare related behavior, leading to more people seeking care when the media coverage is intense [9, 10]. However, there is little research investigating how ED inflow is affected in the following weeks after a terrorist attack.

A terrorist attack is a strain on the society in general and on the health care system in particular. Since ED crowding is a constant problem in many countries, it is important to understand how extreme events like terrorist attacks may impact the inflow of patients to the ED. More importantly, such studies may help us better understand factors and events that influence patients’ decisions to seek medical care at the ED. An understanding of this decision process is of practical value in the planning of ED operations, as well as in the design of the emergency care system in general.

## Materials and methods

### Aims

The objective of this study is to investigate how the terrorist attack affected patient inflow to the emergency departments in close proximity to the attack. While the number of casualties and injured people in the Stockholm terrorist attack could be considered low compared to other recent attacks in Europe and USA [11], it was the largest terrorist attack in Sweden in modern time and received extensive media coverage, both from national and international media [12]. As such, it can be hypothesized that the attack influenced individual’s healthcare seeking decisions. In this study we analyze if, and to what extent, this influence impacted the ED inflow.

### Study design

The study is a retrospective observational study that uses anonymized aggregated data on ED arrivals.

### Study setting and selection of participants

The study was conducted in Stockholm County, Sweden—a region with 2.3 million inhabitants and 26 municipalities. Most inhabitants live in the Stockholm municipality which comprises Stockholm City and its closest surroundings with a population of 1.3 million [13]. The county has seven general hospitals with EDs with a total of approximately 700,000 ED visits per year in 2016 (Table 1). Karolinska Sjukhuset Solna, is the trauma center, hence, severe surgical pediatric cases and major trauma are steered to this hospital ED.

**Table 1:** Emergency Hospitals in Stockholm County. Km = kilometers

Hospital	Emergency census / year (year 2016)	Distance to site of terrorist attack (km)
St Görans sjukhus	86,000	2.4
Södersjukhuset	167,000	2.53
Karolinska sjukhuset Solna	136,000	2.77
Danderyds sjukhus	113,000	6.8
Karolinska sjukhuset Huddinge	86,000	14.4
Södertälje sjukhus	38,000	28.5
Norrtälje sjukhus	27,000	59.2

### Reference collection

Reference literature was collected using PubMed [14], PsycARTICLES [15], reSEARCH [16] and Google Scholar [17]

### Data collection and processing

Data was gathered from the Stockholm County Council data warehouse, VAL [18, 19]. VAL is a comprehensive database consisting of all administrative health care data generated in the Stockholm County on individual level. All health care contacts and the corresponding diagnoses for each visit are stored in VAL, with exception of a few private clinics that operate without subsidies in the Stockholm area [18]. VAL has more than 99% coverage of hospital care in the county [18]. Numbers on daily inflow to all seven hospital bound EDs in Stockholm County were collected and stratified on age for the period of January 1st 2013 –May 31st 2017. The Norrtälje ED (the smallest of the seven EDs) was excluded from the analysis since the surrounding municipality had a different Easter holiday week in 2017 than the rest of Stockholm County. This would make it difficult to disentangle the potential effect of the attack from potential holiday effects. Also, given that the Norrtälje ED is in a rural area more than twice as far away from the attack as the second furthest ED, it is unlikely to have been affected.^1^ Specialized psychiatric EDs were not included.

## Method

To analyze the effect of the terrorist attack on the ED inflow we use the Difference-in-Differences (DiD) estimator, where the control groups are the affected hospitals in the years prior to the terrorist attack. The DiD technique is commonly used to assess treatment effects in observational studies [20–22]. The DiD estimator estimates the effect of the treatment (terrorist attack) by comparing the average change in the outcome (ED inflow) in the treatment group with the average change in the outcome in the control group. This ensures unbiased causal estimates of the treatment effect under the assumption that, without treatment, the treatment and control would have followed parallel trends over time.

To better understand the duration and dynamics of the reduction in ED inflow we use the method outlined by Granger [23] and implemented by Autor [24] to examine how the effect size evolves over time. The technique builds on introducing “leads” and “lags” of the treatment effect. In essence, the ‘leads’ measure the effect of treatment before the treatment (which, of course, is hypothesized to be insignificant), and the ‘lags’ measure the effect of treatment in the periods that follow treatment. The “leads” can be interpreted as placebo effects and the “lags” indicate the duration of the treatment effect.

As outlined in Table 2, we aligned the data from all years in relation to Easter (the terrorist attack occurred one week before Good Friday in 2017, marked in black Table 2). The *before* period is the period where no attack has yet occurred, and the *after* period is the period after the attack has occurred. We eliminated the actual day of the attack from the analysis to exclude effects caused by the acute medical response and the temporary reduction in accessibility, caused by e.g. shut-down of local transportation systems and police barriers, in Stockholm. The *treatment group* includes all six hospitals in 2017 and the *control group* the same hospitals in the years 2013–2016.

**Table 2:**
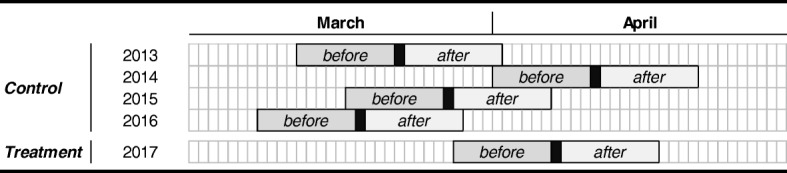
The data in both the control and treatment group are aligned in relation to Easter. The days marked in black in each year corresponds to the Friday one week prior to Good Friday, which is the day of the terrorist attack 2017.

The outcome of interest is the number of patients of each age group arriving at the ED during a given day. To simplify the interpretation of the results, we logarithmized the dependent variable. We also controlled for month, weekday and Easter holiday as well as time-fixed effects on the *age group x hospital* level. To correct for potential correlation within hospitals, the model is estimated with cluster robust standard errors. The regressions are run with the software Stata (version 15, StataCorp LLC, Texas, USA) and the command xtreg.

## Results

### The effect of the Stockholm terrorist attack on ED inflow

Table 3 shows the estimation results for time windows of different lengths (days before and after the attack). The effect of the terrorist attack on ED inflow is given by the *Treatment group x After* coefficients (− 0.0728**, − 0.0703**, − 0.0898***). Since the dependent variable is logarithmized, the coefficients are interpreted as percentages. All three models show a statistically significant relative reduction in ED inflow of 7–9% following the attack. Given an average arrival rate of 1396 patients per day, this is equivalent to a daily absolute reduction of approximately 126 patients for the studied hospitals.

**Table 3:** Difference-in-Differences estimation results. The treatment effect (effect of the terrorist attack) is given by the *Treatment group x After* coefficients and can be interpreted as the absolute percentage decrease in ED visits caused by the attack. The predicted reduction hence ranges from 7.03% (Model 2) to 8.98% (Model 3). The regressions also control for Easter holiday, month, weekday, age group, holiday and age group *x* holiday fixed effects. These coefficients are omitted for the sake of readability

Dependent variable = log(Visits)	Model 1 5 Day Window	Model 2 10 Day Window	Model 3 15 Day Window
Treatment group x After	-0.0728**	-0.0703**	-0.0898***
	(0.0309)	(0.0265)	(0.0197)
Treatment group	0.173**	0.176**	0.176**
	(0.0760)	(0.0793)	(0.0797)
After	0.0139	0.00465	0.0347***
	(0.0125)	(0.0119)	(0.00897)
*Observations*	2430	4860	7290
F Statistic	15.48***	15.03***	18.63***

Note: **p<0.05, ***p<0.01. Cluster robust standard errors in parentheses

The *Treatment group* coefficients indicate an relative increase of 17% in ED inflow in the treatment year (2017) compared to the control years (2013–2016). The *After* coefficient in Model 3 is positive and significant, which shows that, across all years, the after period has slightly higher inflow than the before period. The *Between R-Square* is high compared to the *Within R-Square*, which is typical for fixed effects models, and the *F Statistic* indicate a high overall statistical significance of the regression model.

**Fig. 1:**
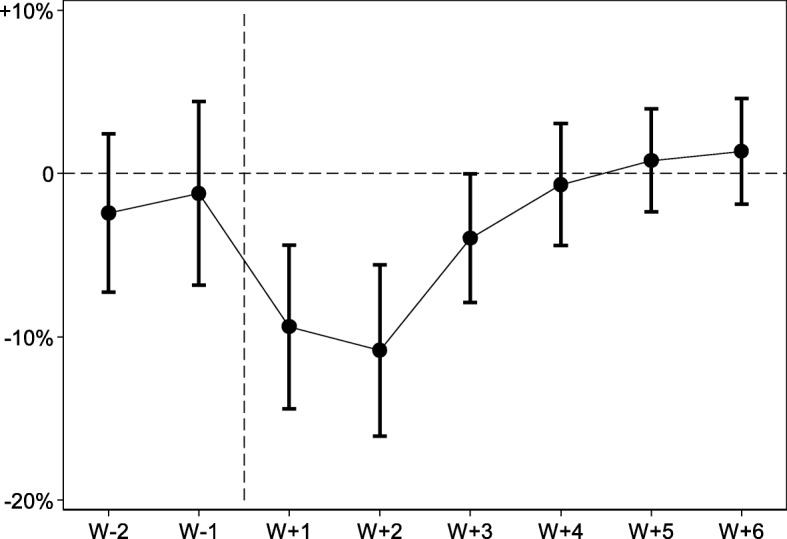
The estimated impact of the terrorist attack on ED inflow over time. Error bars indicate 95% confidence intervals. The graph confirms that no effect occurred prior to the attack (confirmation of the parallel trends assumption). The first and second week following the attack saw an average decrease in ED inflow of around 10% followed by an increase to normal levels in week 3 and forward

Figure 1 shows the results of how effect size evolves over time. In the two weeks preceding the attack there is no significant difference in ED inflow between the control and the treatment groups, which confirms the parallel trends assumption of the DiD estimator. After the attack, the ED inflow significantly decreases in the treatment group during approximately two weeks’ time. Normal levels are regained in week 3. Week 4 to week 6 all exhibit normal levels suggesting that there is no significant “rebound effect”.

### The impact of distance from the attack

The results presented in Table 3 and Fig. 1 show that ED inflow significantly declined following the terrorist attack. We also investigated how this effect was moderated by the distance from the epicenter of the attack. The results are presented in Table 4. The effect of distance on ED inflow are statistically significant, and strong, up to 10 km from the attack but then disappears for EDs that are further away.

**Table 4:** The estimated impact of the terrorist attack on ED inflow for increasing distances from the epicenter of the attack. The Table shows that the effect no longer becomes significant over 10 km distance. Note that the maximum distance in the data is 28.5 km and that the results cannot be extrapolated beyond this point due to the linearity assumption

Distance from attack [km]	Effect size
0	-14.5%***
5	-11.2%***
10	-7.96%***
15	-4.70%
20	-1.40%

Note: ***p<0.01

### Robustness check

Additional analyses were performed as robustness checks of the main results. Data on ED inflow from four hospitals in the two counties adjacent to Stockholm were collected. Since the effect of the terrorist attack diminished with the distance from the epicenter of attack, no significant effect from the terrorist attack was to be expected at hospitals in adjacent regions. The Post-hoc analysis confirmed this. For the first county, Uppsala, arrivals at a single hospital were analyzed. The analysis revealed no significant change in inflow after the attack (− 0.0988, *p*-value: 0.143, *n* = 280). For the second, Södermanland, visits at three hospitals were analyzed. Also, these results did not indicate any significant change in ED inflow (− 0.0546, *p*-value: 0.237, *n* = 1890), despite the relatively large data set.

## Discussion

This study documents a significant decrease in ED patient inflow during the weeks following the Stockholm terrorist attack. The inflow was reduced by 8% during more than two weeks after the terrorist attack compared to the control years. To our knowledge, this is the first study on how general ED attendance is affected in the days and weeks following a terrorist attack. The current results display a different result from previous studies, which conversely point to an increase in the inflow after a terrorist attack [8, 25, 26]. This discrepancy may, in part, be explained by the current study measuring the entire ED inflow rather than a sub-population with specific conditions such as PTSD and other psychiatric disorders [8].

There is no previous work studying the effect of ED inflow in the aftermath of a terrorist attack like the one studied in the article at hand. Similar studies have been made of the hurricane Sandy in the USA 2012. Those studies show an increase of the ED inflow during the period subsequent to the hurricane which would contradict the findings in the study at hand [27, 28]. The two events, though, are inherently different; the hurricane was known in advance and prolonged in time, whereas the terrorist attack bore neither of those characteristics.

Although the results clearly demonstrate that the ED inflow decreased after the terrorist attack, the study does not explain the reasons behind this. While it cannot be ruled out, it must be considered extremely unlikely that 8% of potential patients were healthier during this exact time and in this exact region. A more plausible explanation is that some of the patients that otherwise would have gone to the ED chose not to go during this period. It is possible that the patients did not visit the ED due to fear [29], altruism to avoid congestion at the ED in times of a crisis [4], or unwillingness to visit what could be thought to be a crowded ED. [30] Altmayer et al. has shown that 7.2% of ED visits are best suited for primary care [31]. The observed reduction of ED inflow in the current study is of the same magnitude, which could imply that the decrease in ED inflow represents people that could seek help from other health care providers. The question of whether these 8% actually did seek healthcare elsewhere, and what sort of patients they were, are important questions that we plan to address in future research. A better understanding of the behavior of ED attendees could be useful for the design of health care systems and when deciding actions to reduce ED crowding.

The reduction in patient inflow was seen across all six hospitals, but the effect decreased with the distance from the epicenter of the terrorist attack. Similar distance related differences have been observed in other studies. DiMaggio et al. showed that the number of PTSD related visits to the ED after a terrorist attack was related to the proximity of the hospital to the area of the attack [8]. Similarly, Laugharnea et al. argue that the risk of PTSD is increased by geographic proximity to the attack [32]. Also, the proximity of an indirectly exposed individual (where indirect exposure is defined as *“repeated or extreme exposure to aversive details”*) to the site of an adverse event predicts the severity or amount of symptoms this individual may experience. The effect on ED inflow as a function of the distance to ground zero observed in this study may be a result of this phenomenon [33].

The changes in ED inflow during the study period may also be an effect of the media coverage of the terrorist attack and the considerable amount of indirectly exposed people having psychological symptoms caused by this exposure. The level of media exposure has been shown to predict both the length and severity of psychological symptoms, but the symptoms appear over a limited period of time. This may, in part, explain the unexpected pattern of decreased ED inflow during a period of no more than three weeks seen in this study [33].

**Fig. 2:**
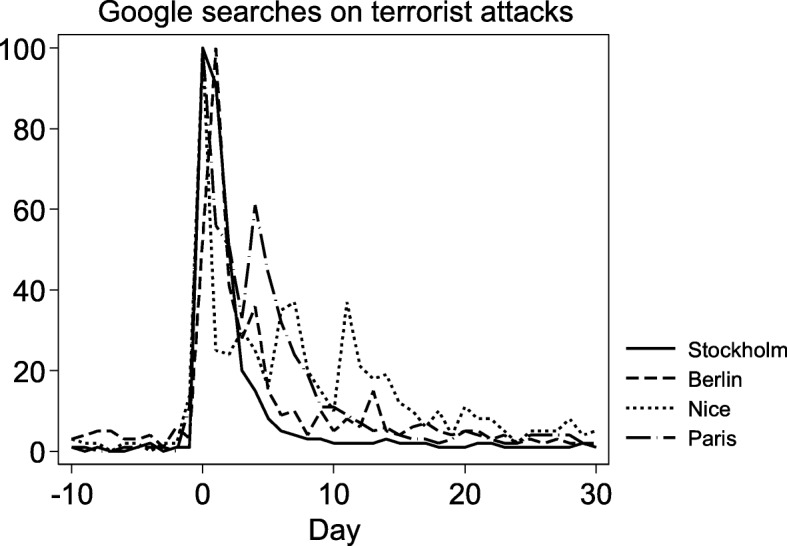
Google searches on terrorist attacks. Google searches on the keywords “terror” and “terroriste”, in conjunction with the location, days before and after four recent terrorist attacks. The attacks included are: Paris 13 November 2015, Nice 14 July 2016, Berlin 19 December 2016 and Stockholm 7 April 2017

An interesting aspect is that effect on ED inflow is strong and significant for two to three weeks and then disappears. This is consistent with Cohen et al.’s study of the World Trade Center attack, which showed that while people show acts of philanthropy and patriotic actions in the direct aftermath of an attack, they will revert to their regular behavior after about three weeks [4]. This “two-week change” in people’s behavior after a terrorist attack is also visible in internet search traffic. Figure 2 shows the volume of Google searches on the keywords “terror” or “terroriste” in conjunction with four recent terrorist attacks in Europe, generated by Google Trends [34]. In previous research, similar searches have been shown to consistently produce more statistically accurate data than other survey- based indicators of public attention and opinion [35–38]. In this case, it is clear that public attention, as measured by the volume of searches, follows the same two-week pattern as the inflow reduction. Whether this public attention is driven by media reporting, a general sense of “moving on”, or some other psychological effect is an open question that merits future studies.

It is well known that ED inflow varies over time. Calendar data (time, day of week, season and holidays) and the number of previous visits have been shown to be the strongest predictors of ED inflow [39, 40]. Though not the primary aim, the current study adds new knowledge by showing how events, e.g. a terrorist attack, can have an effect on ED inflow over a time window of 2–3 weeks.

### Limitations

The study investigates the effect of a single terrorist attack on ED inflow in one county. This may be seen a limitation. However, Stockholm is the most densely populated county in Sweden, and is served by seven large hospital EDs. Therefore, we believe that the results can be generalized to similar urban settings. Moreover, the study includes only aggregated effects on the numbers of visits to the EDs. A detailed understanding of which patients (gender, chief complaints, socioeconomic background etc.) that did not visit the EDs was not analyzed, but is planned for future studies. Neither were visits to psychiatric EDs nor primary care facilities assessed in this study.

The research at hand studies the difference-in-differences effect over a time-period relative to Easter. As Easter may occur during a time period between the end of March and the end of April depending on the year, the dates compared are not the same between years, although weekdays are compared to corresponding weekdays. The variation of ED inflow between March and April is low, why the biasing effect of this conduct is considered to be low.

In addition, the method in this study is that of a retrospective register study and does therefore not explain causation. This being said, a prospective randomized controlled study of this sort of phenomenon, i.e. a terrorist attack, would be difficult to undertake.

## Conclusions

This research shows that ED inflow significantly decreased in the weeks immediately following the Stockholm terrorist attack. This is surprising and raises a number of questions for future research. The results suggest that some patients actively choose when, where and if they should go to the ED. It would be of great value to better understand which type of patients they are, how they make healthcare seeking decisions and where they are best treated.

## Data Availability

The ED inflow datasets analyzed during the current study are not publicly available due to legal issues and county council security but are available from the corresponding author on reasonable request and subsequent vetting by the Stockholm County Council. Data from Google Trends are available from *https://trends.google.com/trends/**.*

## Footnotes

1 Including the Norrtälje ED in the analysis still yields the same qualitative results and conclusions as presented in Table 3.
